# Hemodynamics effects of adrecizumab in sepsis rat

**DOI:** 10.1186/2197-425X-3-S1-A618

**Published:** 2015-10-01

**Authors:** A Blet, M Sadoune, E Polidano, R Merval, C Bernard, JL Samuel, A Mebazaa

**Affiliations:** Inserm, UMR 942 Paris, France; Department of Anesthesiology and Critical Care and Burn Center, GH Lariboisière Saint-Louis, APHP, Paris, France; University Paris VII Denis Diderot, Paris, France

## Introduction

Sepsis and septic shock still represent major health issues, with persisting high morbidity and mortality rates in critically ill patients. Cardiac dysfunction[1] occurs frequently during severe sepsis.

Adrenomedullin (ADM) has been identified as a key mediator in vascular tone regulation[1]. A newly developed anti-ADM antibody Adrecizumab (ADZ) may improve hemodynamic dysfunction during resuscitated murine, cecal ligation and puncture (CLP)-induced septic shock[2].

## Objectives

To determine the beneficial role of ADZ on hemodynamic impairment in a rat model of acute sepsis.

## Methods

For induction of polymicrobial sepsis, cecal ligation and puncture (CLP)[3] was performed in Wistar male rats. ADZ (2 mg/kg) was injected IV 24 h after the surgery. There were 7 animals per group. Invasive blood pressure and cardiac function (by echocardiography) were assessed until 2 hours after ADZ injection. Statistical analysis was performed with 2 ways ANOVA.

## Results

Septic rats had lower mean arterial pressure (MAP) (*p* < 0.0001) 24 h after surgery (at baseline) compared to sham. Septic animas with ADZ had a trend to have a greater MAP. A transient decrease of SF was observed 15 min and 1 h after injection of ADZ (*p* = 0.05). On the other hand cardiac output seems to be increased by ADZ (*p* = 0.61).

## Conclusions

During sepsis in rats, treatment by ADZ seems to have a beneficial effect on cardiac and vascular dysfunction. These preliminary results need to be confirmed in preclinical and clinical studies.

**Figure 1:**
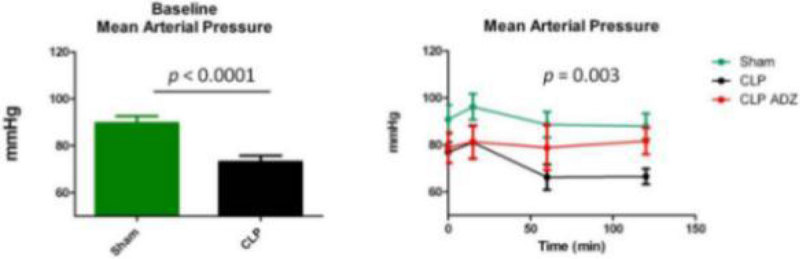
***[Mean arterial pressure]***

## Grant Acknowledgment

Adrenomed
